# Gut microbiota in brain tumors: An emerging crucial player

**DOI:** 10.1111/cns.14081

**Published:** 2023-01-10

**Authors:** Ben Lin, Zhen Ye, Zhao Ye, Meng Wang, Zhan Cao, Renyuan Gao, Yichao Zhang

**Affiliations:** ^1^ Department of Neurosurgery, Huashan Hospital, Shanghai Medical College Fudan University Shanghai China; ^2^ National Center for Neurological Disorders Shanghai China; ^3^ Shanghai Key Laboratory of Brain Function and Restoration and Neural Regeneration Shanghai China; ^4^ Neurosurgical Institute of Fudan University Shanghai China; ^5^ Shanghai Clinical Medical Center of Neurosurgery Shanghai China; ^6^ Department of Endocrinology and Metabolism, Huashan Hospital, Shanghai Medical College Fudan University Shanghai China; ^7^ Department of General Surgery, Shanghai Tenth People's Hospital, School of Medicine Tongji University Shanghai China

**Keywords:** brain tumors, gut–brain axis, microbiota

## Abstract

In recent decades, various roles of the gut microbiota in physiological and pathological conditions have been uncovered. Among the many interacting pathways between the host and gut flora, the gut–brain axis has drawn increasing attention and is generally considered a promising way to understand and treat brain tumors, one of the most lethal neoplasms. In this narrative review, we aimed to unveil and dissect the sophisticated mechanisms by which the gut‐brain axis exerts its influence on brain tumors. Furthermore, we summarized the latest research regarding the gastrointestinal microbial landscape and the effect of gut–brain axis malfunction on different brain tumors. Finally, we outlined the ongoing developing approaches of microbial manipulation and their corresponding research related to neuro‐malignancies. Collectively, we recapitulated the advances in gut microbial alterations along with their potential interactive mechanisms in brain tumors and encouraged increased efforts in this area.

## Graphical abstract

Gut microbiota is a crucial component of the gut–brain axis. Perturbations in gut microbiota composition may set fire to the development of brain tumors. Here in this review, gut–brain axis was classified into three pairs of interactions, including immunological modulation, microbial metabolites‐mediated modulation, and direct invasion. By regulating the secretion of inflammatory factors and immune cell‐mediated responses, microbiota, and tumor cells interact with each other distantly. Microbial metabolites could be transported from gut to the brain via circulation, and thus intestinal metabolic imbalance might augment the overall metabolic imbalance seen in brain tumors. Apart from microbe‐related metabolites, microbe itself could be transported directly through BBB breach via circulation or parasitized immune cells. Specifically, we illustrated the altered microbial landscapes of three most common brain tumors (glioma, meningioma, and pituitary adenoma) along with their potential connections with disease‐specific pathological process.

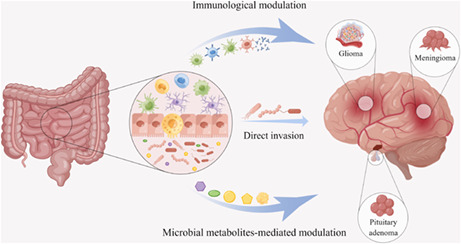

## 1 INTRODUCTION

Central nervous system (CNS) tumors cause a considerable portion of cancer‐related mortality in adults and children.^1^ Recent data have demonstrated that the average annual age‐adjusted incidence rate of all malignant and non‐malignant brain and other CNS tumors was 24.25 per 100,000 people, of which 29.1% are malignant. The most frequently reported type was meningiomas (39.0%), followed by tumors of the pituitary (17.1%), and glioblastoma (14.3%).^2^ Previous studies have identified various genetic alterations that may constitute the molecular basis for these diseases; however, therapies targeting these pathways were either unsuccessful or had minimal survival benefits.^3^

The gut microorganisms comprise the assembly of commensal bacteria, archaea, fungi, and viruses in the gastrointestinal tract, which is referred to as gut microbiome.^4^ Given the diversity among individuals and blurred definition of healthy gut flora, our understanding of the nature and function of the microbiota is still nascent. Nevertheless, as sequencing technology and the utilization of germ‐free (GF) mice advance, the gut microbiota has been demonstrated to play a critical role in physiological activities and pathological conditions.^5^

The gut–brain axis is the bidirectional communication axis between the CNS and gastrointestinal (GI) tract, involving the gastrointestinal microbiota, enteric nervous system, neuroendocrine mediators, autonomic nervous system, and the CNS.^6^ These components function as a multidirectional network that influences biological homeostasis.^7^ Exploring the role of the gut–brain axis in the development or progression of brain tumors may offer novel insights into the molecular etiology of these diseases and provide potential treatment targets.^8^ We hope that this review will promote further studies in this area.

## 2 MECHANISMS UNDERLYING THE GUT‐BRAIN AXIS

**FIGURE 1:**
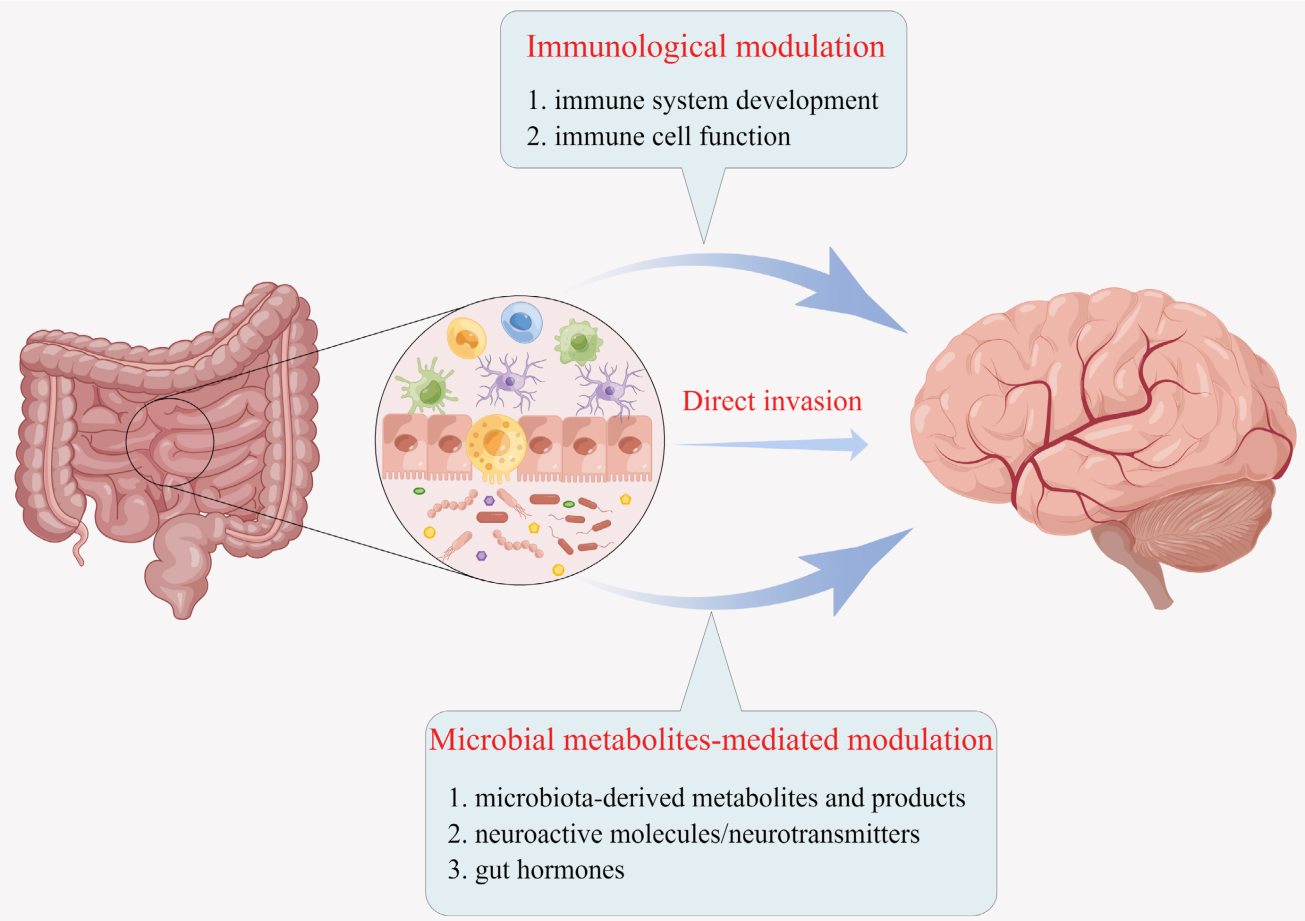
Stratification of the mechanisms involved in the gut–brain axis. Three mechanisms of gut–brain axis were identified, as indicated by the arrows: (1) immunological modulation, (2) microbial metabolites‐mediated modulation, and (3) direct invasion. Gastrointestinal interface between microbiota and host is magnified.

Considerable researches have revealed the intricate networks of the gut–brain axis. Given its substantial influence on the CNS both physically and pathologically, meticulous delineation is warranted.^9^, ^10^, ^11^ Herein, we categorized the mechanisms into three chapters (immunological modulation, microbial metabolites‐mediated modulation, and direct invasion), as shown in Figure 1.

### 2.1 Immunological modulation

#### 2.1.1 Influence on immune system development

Gastrointestinal bacteria have been shown to play an elementary role in the initiation, instruction, regulation, and implementation of the immune system.^12^ Additionally, nearly 70% of all immune cells, comprising lymphoid, and lymphocytes, are located in the intestinal lymphoid tissue.^13^ In the presence of certain gastrointestinal bacteria, dendritic cells mediate the polarization of Th (T helper) cells into Th1 and Th17 cells.^14^ Choi et al. concluded that gut microbiota‐derived molecules might act as antigens in the brain, inducing intestinal Treg cells to migrate to the brains.^15^ These brain‐resident T cells can regulate the fetal‐to‐adult transition of microglia, the primary resident immune cells of the brain.^16^

GF animals develop severe immunodeficiency with impaired intestinal lymphoid architecture and immune functions, indicating that the commensal gut microbiota is essential for the activation and function of immune cells.^17^, ^18^ The commensal *Bacteroides fragilis*‐derived polysaccharide can influence murine immune system maturation by correcting systemic Th1/Th2 imbalance and T‐cell deficiencies in lymphoid tissues.^19^

#### 2.1.2 Effect on immune cell function

Peripheral immune cells are influenced by the gut microbiota. GF mice have fewer proinflammatory Th17 cells in the lamina propria of their gastrointestinal tract than specific pathogen‐free controls.^20^ Furthermore, several bacterial species (e.g., *Lactobacilli*, *Proteobacteria*, *Clostridium difficile*, *Enterococci*, *Bacteroides fragilis*) are known to stimulate specific pro‐ and antiinflammatory immune cell populations.^21^ For example, *Bacteroides fragilis* can induce the differentiation of interleukin‐10 (IL‐10)‐secreting T regulatory cells (Tregs),^22^ which impairs anticancer Th1 immunity and promotes glioma progression and aggressiveness.^23^ Furthermore, particular microbiota can drive immune suppression, which is commonly observed in malignancies, including glioblastomas.^24^

The CNS was once considered an immune‐privileged site; however, the permeable nature of the brain–blood barrier (BBB) and the recent discovery of functional lymphatic vasculature indicate that immune cells play a physiological and pathological role in the CNS.^25^, ^26^ In addition to glial cells, resident immune cells (e.g., macrophages, CD8+ T cells, Tregs, and other CD4+ Th cells) actively engage in innate and adaptive immune responses.^27^

Among these subsets, microglia, which migrate to the brain during development and self‐renew until adulthood, are well‐studied.^28^ Apart from protecting the brain from pathological conditions, microglia can regulate synaptic transmission, synaptic pruning, and neuronal circuit formation.^29^, ^30^ The microbiome influences microglial properties and function. In GF mice, microglia exhibit altered morphology and transcriptomic profiles, accompanied by inhibition of the maturation state, as indicated by increased immature microglia in the brain cortex.^31^ Similarly, antibiotic‐treated mice present with increased naïve microglia, although the total number of microglia remains unchanged.^31^, ^32^ Immature microglia in GF mice tend to display impaired immune activation and responses to challenges, accompanied by reduced inflammatory factors and inhibited innate immune signaling.^8^, ^31^ Remarkably, the microbial deficiency‐induced immunosuppressive phenotype in GF mice can be reversed by postnatal administration of microbial short chain fatty acids (SCFAs), suggesting that certain microbial species and metabolites are involved in the maturation and homeostasis of microglia. One study revealed that increased intestinal *Ruminococcus* induced the antiinflammatory polarization of microglia and attenuated neuronal degeneration and necrosis caused by epilepsy in LPS‐induced mice model.^33^ Moreover, GPR43 in innate immune cells mediates inflammatory responses by interacting with SCFAs, and mice with GPR43 deficits display severe morphological defects in microglia, similar to the defects observed in GF mice.^31^ Since GPR43 is intimately related to inflammasomes, the interplay among them may contribute to maintaining microglia‐mediated immunological homeostasis.

Chronic inflammation and an imbalanced immune environment have long been associated with oncogenesis and tumor progression. For example, a high antiinflammatory/pro‐inflammatory ratio is found in glioblastoma (GBM) and correlates with poor survival, and antiinflammatory cells can secrete IL‐10, epidermal growth factor (EGF), and vascular endothelial growth factor (VEGF).^34^ Interestingly, gut microbiota‐derived metabolites can drive microglial polarization towards antiinflammatory phenotype^35^ and Tregs can be promoted by diet‐derived SCFAs.^36^ Tregs can exacerbate the immunosuppressive microenvironment by producing IL‐10 and transforming growth factor‐β (TGF‐β).^17^ Accordingly, Treg levels are correlated with brain tumor grade, and in vivo depletion of Tregs improves survival in mice.^37^ Besides, previous research showed that microbiome‐mediated inflammatory reactions exist in central and peripheral organs, probably serving as a disease biomarker and a therapeutic target for stroke.^38^ With respect to brain tumors, inspections of the inflammatory response in peripheral organs and the potential involvement of gut microbiome should be conducted in future research.

The nuclear factor kappa‐B (NF‐κB) pathway plays a substantial role in the production of inflammatory cytokines such as IL‐6 and IL‐8, thereby regulating the tumor microenvironment. In cancer cells, abnormalities of the NF‐κB pathway activate survival genes. Specifically, IL‐6 can induce NF‐κB in GBM, resulting in the activation of signal transducer and activator of transcription 3 (STAT3) and increased tumor aggressiveness.^39^ High NF‐κB expression is associated with poor survival in mesenchymal GBM.^40^ Given that circulating SCFAs can enter the CNS especially when the BBB is disrupted,^41^ it is reasonable to speculate that gut microbiota‐related metabolites, such as SCFAs, could influence the NF‐κB function of cancer and immune cells inside the brain.

In conclusion, the gut flora is critical for the establishment of global and intracranial immune environments, and the functional status of peripheral along with intracranial immune cells. Meanwhile, a suppressive immune microenvironment influences all stages of brain cancer development, contributes to tumor initiation, and facilitates tumor cells to evade immune surveillance, probably involving the gut microbiota.

### 2.2 Microbial metabolites‐mediated modulation

#### 2.2.1 Microbiota‐derived metabolites and products

Microbial‐derived metabolites and products are major contributors to the microbiota–gut–brain axis, exerting their effects primarily via receptor‐mediated interactions in host tissues or cells. Among them, SCFAs and endogenous tryptophan are the most well‐documented metabolites. SCFAs, derived from microbial decomposition of carbohydrates, are related to glucose homeostasis, mucosal serotonin release, lymphocyte function, learning, and memory acquisition via the preservation of brain integrity.^42^, ^43^, ^44^ As mentioned above, some circulating SCFAs can enter the CNS. GF mice have been documented as having increased BBB permeability. Recolonization of the same mice with SCFA‐producing bacteria recovered BBB integrity, which further supports the role of SCFAs in CNS homeostasis.^45^ Mechanistically, studies have reported interactions between SCFAs and G‐protein coupled receptors (GPR), such as GPR41 and GPR43,^46^ potentially serving as a connection between SCFA and the CNS. On the other hand, SCFAs, as illustrated above, can modulate the immune system. Mechanistically, SCFAs can influence metabolism and inhibition of histone deacetylases (HDACs), thereby modifying histone acetylation.^47^ The suppression of histone deacetylase activity inhibits the activities of tumor necrosis factor‐α (TNF‐α) and NF‐κB.^48^ Further, the chemokine pattern of dendritic cells (DC) is regulated by SCFAs such as acetate and propionate,^49^ and neutrophils, B cells, and T cells can also be modulated by SCFAs.^50^

The gut microbiota can directly convert tryptophan into metabolites with immunomodulatory capacity (e.g., indolic acids, indole, and tryptamines).^51^ Certain members of the *Lactobacillus* genus, capable of metabolizing tryptophan, were recently identified to play critical roles in the activation of aryl hydrocarbon receptors and consequently affect cell cycle regulation and T‐cell differentiation.^52^ Furthermore, studies have demonstrated the effect of dietary tryptophan on proinflammatory T‐cell responses, thereby inducing CNS autoimmunity.^53^

Apart from the immunoregulatory effect, Westfall et al. also discovered antidepression and antianxiety benefits of microbiota‐derived metabolites from the combination of probiotics and polyphenol‐rich prebiotics. These metabolites can act as the ligand for aryl hydrocarbon receptor (AHR) and reprogram the ratio of T‐cell subsets, which may alleviate chronic stress induced inflammatory responses in prefrontal cortex.^54^

The host metabolic status affects the microbial composition, and microbial metabolites can induce epigenetic modifications^55^; for example, *Brecibacteruim* spp regulates the balance of ⍺‐ketoglutarate (⍺KG) and glutamate.^56^ The IDH1/2 mutation in glioma inhibits multiple ⍺KG‐dependent enzymes, leading to aberrant DNA methylation. Thus, the gut microbiota and its derived metabolites might modify the epigenetic status, contributing to glioma progression.^57^ Microbiota‐derived SCFAs also regulate neuronal, glial, and tumoral epigenetics. Acetate participates in acetyl‐CoA production and subsequently affects the acetylation of rapamycin‐insensitive companion of mTOR (RICTOR), potentially promoting glioma tumorigenesis.^58^

Besides, accumulating evidence reveals that the circadian rhythm has an impact on glioma pathophysiology, and the internal characteristics concerning the circadian clock in glioma involve stemness, metabolism, radiotherapy sensitivity, and chemotherapy sensitivity.^59^ Meanwhile, alterations of microbiome‐derived metabolites such as ergothioneine were observed to be associated with circadian function.^60^ Yet, it remains to be addressed whether the gut microbiota may affect tumorigenesis through disruption of circadian rhythm.

In addition to microbial metabolites, microbiota‐derived products also play major roles in the microbiota–gut–brain axis through interactions with toll‐like receptors (TLRs) in the enteric nervous system (ENS) and CNS. For example, lipopolysaccharide (LPS), released by gram‐negative bacteria are recognized by TLRs expressed on CNS microglia, subsequently inducing proinflammatory cytokine production and proliferation.^61^ Remarkably, this immune response has been found to result in neuroinflammation, microglial activation, and neuronal cell loss, triggering cognitive impairments, anxiety, and depression.^62^ Polysaccharide A, another common microbial product, is secreted by *B. fragilis* and can be recognized by TLRs,^5^ provoking a protective CNS antiinflammation response.^63^

#### 2.2.2 Neuroactive molecules/neurotransmitters

Aside from metabolites and products, bacteria produce a variety of major neurotransmitters such as dopamine, noradrenaline, serotonin, gamma‐aminobutyric acid (GABA), acetylcholine, histamine, and tryptophan metabolites, sustaining the gut‐brain axis.^64^, ^65^ Serotonin, GABA and tryptophan metabolites cannot directly affect the CNS, owing to the inability to cross the BBB but can influence the nervous system by interacting with cells in the ENS.^46^

Gut microbes also regulate the abundance of host neurotransmitters such as dopamine, norepinephrine, and serotonin.^66^ Studies in GF mice have demonstrated that the absence of microbiota modulates the neurotransmitter turnover in the CNS and ENS. For example, GF mice display increased turnover rates for dopamine and norepinephrine in the brain.^67^ A combination of 46 *Clostridium* species was shown to restore dopamine and norepinephrine levels in the cecal lumen of GF mice.^68^ However, whether this effect is due to direct production of neurotransmitters or results from the modulation of host production remains unclear. Similarly, gavage of *Enterococcus faecium* and *Lactobacillus rhamnosus* in young mice increased brain dopamine levels.^69^ Moreover, the gut microbiota can produce certain cofactors such as tetrahydrobiopterin (BH4), thereby promoting tyrosine hydroxylase (TH) activity in the brain, consequently leading to increased dopamine levels.^70^

GF mice display an increased turnover rate of serotonin in the brain,^67^ and substantially decreased peripheral serotonin compared with control mice.^71^ However, studies investigating the influence of microbiota on serotonin using GF mice obtained inconsistent results; one reported increased turnover and unchanged levels of serotonin in the striatum,^67^ while another showed increased levels of both serotonin and 5‐hydroxyindoleacetic acid (5‐HIAA) in the hippocampal regions.^72^ Correspondingly, supplementation of *Lactobacillus plantarum* to GF mice significantly increased serotonin and dopamine levels in the striatum.^73^

The gut microbiota also affects the circulating GABA, with GF animals demonstrating reduced GABA levels in the gut lumen and serum.^74^ The administration of *Lactobacillus rhamnosus* increased brain GABA level^75^ and alleviated depressive and anxiety‐like behaviors, along with alterations in the transcriptomic profile related to cerebral GABA receptors.^76^ Furthermore, a recent study showed that *Lactobacillus rhamnosus JB‐1* supplementation increased brain GABA and glutamate/glutamine levels, indicating that the gut microbiota might regulate glutamate production pathways in the brain.^75^, ^77^

Despite the interactions mentioned above, how these neuroactive molecules affect the CNS remains unclear.

#### 2.2.3 Gut hormones

Enteroendocrine cells (EECs), which constitute the interface between the gut microbiota and host, are modulated by the diversity and composition of the gut bacteria, and thus generate fluctuations in secreted hormones, consequently participating in gut–brain crosstalk.^78^ It has been shown that bacterial metabolites (e.g., LPS, SCFAs, and tryptophan) can stimulate EECs of the gut epithelium to produce neuropeptides (peptide YY, neuropeptide Y, cholecystokinin, glucagon‐like peptide (GLP)‐1 & 2, and substance P). These neuropeptides enter the bloodstream to reach local receptors and influence ENS neurons and the vagal nerve.^79^ Additionally, the gut microbiota may modulate the production of neuromodulators, as evidenced by increased levels of GLP‐1, corticosterone, and adrenocorticosterone in GF mice.^66^ Collectively, these results suggest that the gut microbiota regulate the release of gut hormones from EECs through metabolites or bacterial components.

Notably, gut hormones also affect the microbiota, altering the microbial profile of the gut.^80^ Future studies are warranted to determine the cause and effect of the gut hormone‐gut microbiota association.

### 2.3 Direct invasion

Although the detailed mechanisms remain poorly understood, direct microbial invasion beyond the BBB is possible. Regardless of heterogeneous requirements, common prerequisites for invasion include asymptomatic colonization of host mucosal surfaces, sustained survival in the bloodstream, resistance or escape from immune responses, and breaching the BBB through various pathways.^81^ During tumor development, disorganized and leaky vasculature may allow entry of circulating bacteria, for which the immunosuppressed environment may provide a refuge.^82^ Indeed, recent studies have confirmed the presence of intratumor microbiome in brain tumors^83^, ^84^; however, the detailed mechanisms underlying this direct host–bacteria interaction remain to be clarified.

## 3 ABERRANT MICROBIAL LANDSCAPES AND DYSREGULATED MICROBIOTA–GUT–BRAIN AXIS IN BRAIN TUMORS

**FIGURE 2:**
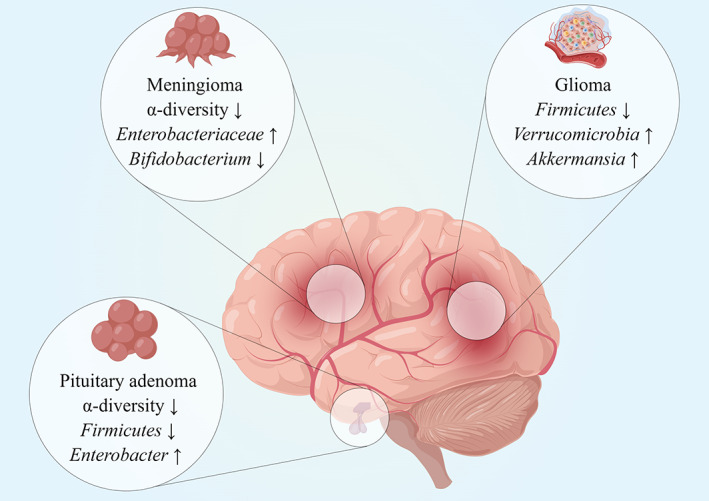
Brain–tumor‐specific alterations in gut microbial profiles. Currently, several studies have reported variations in microbial constructions of glioma, pituitary adenoma, and meningioma, respectively. Disease‐specific changes are concisely presented in the corresponding magnifiers.

Studies focusing on brain lesions specific alterations in gut microbiota have emerged in recent years, with most focusing on the three most common brain tumors, glioma, pituitary adenoma, and meningioma. The aforementioned changes have been summarized and described in Figure 2, and detailed variations are listed in Table 1.

**TABLE 1:** Summary of the current studies describing microbiota‐related alterations in brain tumors

	Subjects	Treatment	Increased	Decreased
H. Kim et al.	C6 glioma cells	Compound K	—	Phosphorylation of PKCα and ERK1/2, MMP9, MMP2, and cell migration
A. Patrizz et al.	Mouse glioma model	—	*Verrucomicrobia* phylum, *Akkermansia* genus	*Firmicutes* phylum, *Firmicutes/Bacteroides* ratio
		Temozolomide	—	—
	Glioma patients	—	*Bacteroidetes*, *Proteobacteria*, *Verrucomicrobia*, *Akkermansiaceae* and *Akkermansia* (IDH‐WT)	*Firmicutes/Bacteroides* ratio (both IDH‐WT and IDH‐Mut); *Firmicutes* and *Actinobacteria* (IDH‐WT)
		Temozolomide	—	*Verrucomicrobia*, *Akkermansiaceae*, and *Akkermansia*
A. Dono et al.	Mouse glioma model	—	Serotonin 3‐methyl valerate, caproate and acetylcholine; *Verrucomicrobia*, *Bacteroides*, and *Akkermansia*	Dihydroxy phenyl acetic acid, adenosine, histamine, butyrate, propionate, acetate, norepinephrine, 5‐hydroxyindoleacetic acid, GABA, tryptophan, valerate, and aspartic acid
		Temozolomide	Acetylcholine, 3‐methyl valerate, caproate	Histamine
	Glioma patients	—	—	5‐Hydroxyindoleacetic acid, and norepinephrine
		Chemoradiotherapy	—	—
G. D'Alessandro et al.	Mouse glioma model	Antibiotics	*Burkholderiales*, arg1, p2ry12, and inos mRNA expression and ARG1 and P2RY12 proteins in microglia	Species diversity and α‐diversity, *Prevotellaceae*, *Rikenellacaea*, and *Helicobacteraceae*, total peripheral NK cells number, frequency of CD27+/CD11b+ cell subset
X. Li et al.	Mouse glioma model	—	*Intestinimonas*, *Lactobacillus*	*Anaerotruncus*
		Temozolomide	*Intestinimonas*, *Akkermansia*, *Bifidobacterium*	*Anaerotruncus*
L. Wang et al.	Mouse glioma model	Oral gavage of probiotics	PTEN protein and mRNA	*Staphylococcus*, *Helicobacter* and p‐PI3K protein expression, survivin protein, and mRNA expression
K. Dees et al.	Mouse glioma model with humanized microbiome	Anti‐PD‐1 responder VS anti‐PD‐1 non‐responder	*Bacteroides cellulosilyticus*, *Alistipes indistinctus*, *Blautia hydrogenotrophica*, and *Eubacterium limosum*; CD8+ and CD4+ T‐cells producing IFN‐γ, CD8+/Treg ratio	—
J. Zhu et al.	Recurrent malignant gliomas patients	Bevacizumab + Temozolomide vs. Temozolomide	*Actinobacteria*, *Firmicutes*, *Bacteroidetes*	*Proteobacteria* and *Cyanobacteria*
Y. Fan et al.	Mouse glioma model		*Firmicutes*, *Firmicutes/Bacteroidetes* ratio	*Bacteroidia*, *Actinobacteria* and *Bacteroidetes*, Foxp3 expression in the brain
		Antibiotics vs. No antibiotics	Tumor progression	*Bacteroides* and *Firmicutes*; Foxp3 expression in brain tissues
		Antibiotics + FMT VS Antibiotics + NaCl	*Bacteroidetes*; Foxp3 expression in brain tissues	*Proteobacteria*, *Rickettsiales*, *Alcaligenaceae*, *Sutterella*, and *Flexispira*; tumor development rate
H. Jiang et al.	Glioma and meningioma patients		*Enterobacteriaceae* (meningioma); *Fusobacterium* and *Akkermansia* (glioma)	α‐Diversity, *Firmicutes/Bacteroidetes* ratio; *Lachnospira*, *Agathobacter*, and *Bifidobacterium*; metabolism of D‐Glutamine and D‐glutamate, nucleotide excision repair (NER), and endocytosis
H. Aglae et al.	Mouse glioma model		Lithocholic acid	Butyrate, isobutyrate, propionate and valerate
A. Hacioglu et al.	Acromegaly patients		*Bacteroidetes*, *Bacteroides*	α‐Diversity, *Firmicutes*, *Firmicutes/Bacteroidetes* ratio, *Bifidobacterium*, *Collinsella*, *Butyricimonas*, *Clostridium*, *Oscillospira*, and *Dialister*
D. Nie et al.	GHPA and NFPA patients		*Enterococcus*, *Prevotella*, and *Bifidobacterium*, predicted function related to immune system (GHPA)	*Escherichia‐Shigella*, *Faecalibacterium*, and *Megamonas*
	Tumor‐bearing nude mice	FMT from GHPA donors	Tumor weight and volume; number of PD‐L1 positive cells and extent of infiltration of CD8+ cells (tumor tissues); number of CD3+CD8+ cells and level of sPD‐L1 (blood)	—
B. Lin et al.	GHPA patients		*Oscillibacter* and *Enterobacter*; *Alistipes shahii*, *Odoribacter splanchnicus*, *Prevotella stercorea*, *Enterobacter* sp. *DC1* and *Enterobacter* sp. *940 PEND*; amino acid metabolism pathway	*Blautia* and *Romboutsia*; *Carnobacterium* sp. *N15 MGS 207*, *Phascolarctobacterium* sp. *CAG 207*, and *Bacteroides* sp. *3_1_19*; carbohydrate metabolism pathway
J. Hu et al.	PA patients		*Clostridium innocuum*	*Oscillibacter* sp. *57_20* and *Fusobacterium mortiferum*
S. Sahin et al.	Acromegaly patients		*Alphaproteobacteria*, *Proteobacteria* and *TM7; Bacteroides*, *Escherichia*, and *Lactobacillaceae*	*Firmicutes/Bacteroidetes* ratio, *Clostridiales, Dialister*, *Veillonellaceae*

### 3.1 Glioma

Glioma is the most common primary malignant tumor of the CNS in adults, of which the majority (58.4%) are glioblastoma multiforme (GBM), the most aggressive form.^2^ GBM is marked by high cell proliferation, active angiogenesis, and vigorous invasion. Despite multimodal therapy (surgery, chemotherapy, and radiotherapy), the median survival time (14–15 months) remains unsatisfactory. Although state‐of‐art therapies such as immunotherapy are emerging, many barriers remained unsolved.^85^ Hence, further research expanding the field of tumor cell–host interactions to identify potential therapeutic targets is required.

#### 3.1.1 Alterations of the gastrointestinal microbial ecosystem

The first study implying the link between gut microbiota and glioma dated back to 2016 when Kim et al. showed that the gut microbiota‐derived metabolite, compound K, decreased phosphorylation of PKCα and ERK1/2, expression of MMP9 and MMP2, and subsequent cell migration in glioma cells.^86^ Since then, studies focusing on glioma‐specific gastrointestinal microbial alterations have flourished. In 2020, a preliminary study by Patrizz et al. demonstrated a consistent trend of enriched (*Verrucomicrobia* and *Akkermansia*) and depleted taxa (*Firmicutes* and subsequent *Firmicutes/Bacteroides* ratio) in both mouse glioma models and glioma patients,^87^ which can be reversed by temozolomide, a common chemotherapeutic agent. Further research showed similar results in mouse models^88^ and glioma patients.^89^ Mechanistically, these studies proposed that *Akkermansia* degrades the intestinal mucosal layer to induce inflammatory responses, neurotoxicity, and BBB disruption in the glioma microenvironment, and temozolomide‐associated alteration is induced by glioma control rather than the chemotherapeutic agent itself. However, other studies led to different results; for example, Fan et al. reported increased *Firmicutes*, *Firmicutes/Bacteroides* ratio combined with decreased *Bacteroidia*, *Actinobacteria*, and *Bacteroidetes*.^90^ Li et al. detected increased *Intestinimonas* and *Lactobacillus* along with decreased *Anaerotruncus*, and elevated abundance of *Akkermansia* as well as *Bifidobacterium* after temozolomide treatment, suggesting that induction of *Akkermansia* and *Bifidobacterium* may contribute to the antitumor effect.^91^ Another study reported that coadministration of *B. lactis* and *L. plantarum* could inhibit glioma growth via PI3K/AKT pathway and regulate gut microbiota composition and metabolites in mice.^92^ This heterogeneity in results may be due to limited sample sizes and different ethnicities of enrolled participants.

#### 3.1.2 Dysregulated gut‐brain axis components

The microbiota has been shown to influence the status of natural immunity. Alessandro et al. demonstrated increased expressions of certain genes (*arg1*, *p2ry12*, and *inos*) and specific proteins (ARG1 and P2RY12) in microglia, combined with decreased total peripheral natural killer (NK) cells and frequency of CD27+/CD11b + cell subset.^93^ Microbiota manipulation could affect glioma progression and induce fluctuation of Foxp3 (a signature of Tregs) expression,^90^ indicating that the gut microbiota may influence adaptive immunity and regulate glioma progression. Furthermore, microbial constructions may affect the efficacy of antiprogrammed cell death protein 1 (PD‐1), the most well‐known immunotherapeutic agent. In mouse‐glioma models with a humanized microbiome, Dees et al. uncovered increased taxa (*Bacteroides cellulosilyticus, Alistipes indistinctus, Blautia hydrogenotrophica*, and *Eubacterium limosum*), expanded CD8+ as well as CD4+ T‐cells, and higher CD8+/Treg ratio in those who responded to anti‐PD‐1 therapy, showing the potential of the microbiota as an indicator guiding immune therapy.^94^

Aside from the involvement of immunological modulation, altered microbial metabolism should be discussed in the context of the dysregulated gut‐brain axis in the glioma. For example, Dono et al. discovered that SCFAs and neurotransmitters (e.g., butyrate, propionate, acetate, norepinephrine, and 5‐HIAA) were decreased following glioma growth in mouse‐glioma model, and a similar trend was detected in both tumor‐bearing mice and glioma patients, which was normalized by chemotherapy.^88^ Notably, a tricyclic antidepressant‐induced increase of norepinephrine is implicated in limiting glioma growth,^95^ and decreased norepinephrine may be associated with glioma growth. In another study focusing on late‐stage glioma associated mortality, Aglae et al. reported decreased levels of SCFA (butyrate, isobutyrate, propionate, and valerate) and increased levels of lithocholic acid, and demonstrated that these alterations were involved in end‐stage disease progression.^96^

### 3.2 Meningioma

Meningiomas are the most common primary intracranial tumor in adults, accounting for ~39.0% of all brain tumors and 54.5% of non‐malignant tumors. Meningioma has a lower five‐year relative survival (88.2%) than other non‐malignant tumors. Aside from surgical treatment, no effective medical therapy for meningioma patients exists, and new treatments are limited by poor understanding of tumor biology.^2^

#### 3.2.1 Alterations of the gastrointestinal microbial ecosystem

To our knowledge, only one study has investigated the microbial alteration in meningioma patients. In the brain tumor cohort enrolled by Jiang et al., α‐diversity, *Firmicutes/Bacteroidetes* ratio, and abundance of *Lachnospira*, *Agathobacter* as well as *Bifidobacterium* were decreased in meningioma patients, while *Enterobacteriaceae* were enriched. The researcher inferred that this imbalance in microbial profiles contributes to the dysbiosis of immune and intestinal environments, and even promotes chronic neurological inflammation through neurotoxicity.^89^

#### 3.2.2 Dysregulated gut–brain axis components

The same study reported the downregulation of genes related to the metabolism of D‐glutamine and D‐glutamate, nucleotide excision repair (NER), and endocytosis, likely affecting inflammatory and immune responses of the meningioma patients.^89^

### 3.3 Pituitary adenoma

Pituitary adenomas comprise approximately 17.1% of intracranial tumors.^2^ Management of these benign tumors requires prompt diagnosis and multidisciplinary treatment of local mass effects and peripheral endocrinopathies.^97^ Because tumors are characterized by excessive levels of different hormones, the clinical manifestations and corresponding management vary widely.

Among different subtypes of pituitary tumors, growth hormone‐secreting pituitary adenoma (GHPA) causes acromegaly, with an incidence of ~10 cases per 1 million persons.^98^ Excessive growth hormone (GH) and insulin‐like growth factor 1 (IGF‐1) levels induce remarkable somatic dysmorphism and metabolic dysfunctions.

#### 3.3.1 Alterations of the gastrointestinal microbial ecosystem

Studies regarding microbial alterations in pituitary adenomas have been recently conducted. Hacioglu et al. reported decreased microbial diversity, *Firmicutes* and *Firmicutes/Bacteroidetes* ratio while increased *Bacteroidetes* along with *Bacteroides* in acromegaly patients.^99^ The authors postulated that the imbalanced *Firmicutes/Bacteroidetes* ratio may induce inflammation and deficiency of beneficial metabolites, such as butyrate. However, later research conducted in a different ethnicity found different species that were increased (*Enterococcus*, *Prevotella*, and *Bifidobacterium*) and decreased (*Faecalibacterium* and *Megamonas*).^100^ Due to the limitations of 16 S rRNA amplicon sequencing, our group further utilized the metagenomic sequencing in GHPA patients and observed increased abundance of *Oscillibacter* and *Enterobacter*, along with decreased abundance of *Blautia* and *Romboutsia*.^101^ We further observed a positive correlation between *Enterobacter* and GH/IGF‐1 axis, implying the probable involvement of microbial variation in the pathophysiological process. Additionally, Hu et al. used metagenomic sequencing to study the differences in the microbial diversity between the invasive pituitary adenoma (IPA) and noninvasive pituitary adenoma (NIPA). They discovered that *Clostridium innocuum* was enriched in both subtypes of pituitary adenoma compared with the healthy controls, whereas *Oscillibacter* sp. *57_20* and *Fusobacterium mortiferum* were enriched in normal individuals.^102^

#### 3.3.2 Dysregulated gut–brain axis components

Nie et al. hypothesized that the microbial profile in GHPA patients is related to the immune system. They found that fecal microbiota transplantation (FMT) from GHPA donors in tumor‐bearing nude mice increased tumor weight and volume, expanded PD‐L1 positive cell populations and CD8+ cell infiltration in tumor tissues, and increased levels of CD3 + CD8+ cell and sPD‐L1 in the peripheral circulation. This indicates that GHPA‐specific flora influences the status of the immune system, which may facilitate the escape of tumor cells from immune surveillance.^100^

Regarding abnormal microbial metabolites‐mediated modulation, our group observed enhanced expression of genes related to amino acid metabolism and decreased abundance of genes in carbohydrate metabolism.^101^ Since amino acid and carbohydrate metabolic pathways are involved in the production of short‐chain fatty acids (SCFA),^103^ the altered metabolic profiles of gastrointestinal microbiota in GHPA patients may indicate variations in SCFA metabolism and downstream inflammatory status. Further studies are needed to elucidate the specific underlying mechanisms.

Given that several studies have suggested a close interaction between the gut–brain axis and hypothalamus–pituitary–adrenal axis,^104^, ^105^, ^106^ it is rational to speculate that the gut microbiota may play a significant role in the pathological process of Cushing's disease (a subtype of pituitary adenomas). Further research is needed to clarify this hypothesis.

### 3.4 Others

The scope of neuro‐oncology is much wider than that of the brain tumor types covered in this review, with CNS lymphoma, neurofibroma, metastatic tumors, etc. However, there are currently no studies exploring their specific gut microbiota alterations. Hence, we call for future studies to inspect these disease‐specific microbial profiles.

## 4 LEVERAGING MICROBIAL MANIPULATION AS POTENTIAL ADJUVANT TREATMENT

### 4.1 Fecal microbiota transplantation (FMT)

FMT can ameliorate neurological diseases, such as seizures.^107^ Nevertheless, merits of FMT need to be tested further because of the unclear definitions of what constitutes a favorable microbiota and uncertainty regarding the benefits and long‐term effects. To address this, several clinical trials have been launched.^108^ Thus, the extended utilization of FMT to alleviate various diseases is still hindered by its lack of specificity.

For GBM, the immunosuppressive microenvironment has constrained therapeutic success. Balancing the gut microbiota can reduce immune suppression in the microenvironment around GBM. Immune therapy generally involves immune checkpoint antibodies, of which the most famous is PD‐L1. PD‐L1 blockade shows enhanced antitumor effects when used in conjunction with *Bifidobacterium*. In mice with melanoma, *Bifidobacterium* administration ameliorated tumor growth by influencing the immune microenvironment, promoting DCs maturation, CD8+ T‐cell stimulation and immune cell recruitment.^109^ FMT from PD‐L1 therapy responders into germ‐free mice improved PD‐L1 therapeutic efficacy,^110^ while PD‐1 blockade combined with antibiotic treatment impairs treatment efficacy and overall survival.^111^ Recent studies have found that mice treated with anticytotoxic T‐lymphocyte‐associated protein 4 (CTLA‐4) antibodies consistently present T‐cell‐mediated mucosal injury, resulting in alterations of the composition of gut flora. Oral administration of *B. thetaiotaomicron* or *B. fragilis* to microbiota‐depleted mice facilitates the response to anti‐CTLA‐4 treatment by inducing a Th1 response in the tumor‐draining lymph nodes.^112^ These advances in understanding the effect of microbiota on other cancers may offer alternatives for brain cancer treatment.

### 4.2 Probiotics

Probiotics are live microorganisms which exert health benefits when consumed or applied to the body. They are found in yogurt, fermented foods, and dietary supplements. Modified probiotics, genetically engineered to maximize the beneficial effects, have been introduced and included in more traditional pharmaceutical routes.^44^, ^113^, ^114^

In recent years, probiotic administration has been shown to regulate neuroendocrine homeostasis. For example, in a randomized, double‐blind, placebo‐controlled study, *Lactobacillus plantarum 299v* was administered to 41 students with an upcoming examination, subsequently showing a correlation with decreased corticosterone levels.^105^ Similarly, a probiotic formulation comprising *L. helveticus R0052* and *B. longum R0175* decreased median urinary free cortisol levels and psychological distress in a double‐blind, controlled, randomized, parallel study.^106^ Likewise, *B. longum 1714* supplementation to healthy male volunteers in a placebo‐controlled study attenuated increases in cortisol and subjective anxiety, in parallel with improvements in hippocampus‐dependent visuospatial memory performance and changes in brain activity on electroencephalography.^104^ In conclusion, manipulation of the gut microbiota regulates the neuroendocrine system, implying a potential application in the management of endocrinopathies in pituitary tumors. However, heterogeneity in composition, stability, and authenticity, lack of consensus on dosage, duration, and specific strains to use, and host colonization resistance must be resolved before expanding the application of probiotic‐based therapies to brain tumors.

### 4.3 Antibiotics

Manipulating gut microbiota through antibiotics also deserves additional attention. For instance, vancomycin was reported to enhance the efficacy of CTLA‐4 blockade therapy by decreasing harmful gram‐positive bacteria without affecting gram‐negative *Burkholderiales* and *Bacteroidales*.^112^ Further, another preclinical study showed that vancomycin could potentiate the antitumor effect of radiotherapy in mouse models (melanoma, lung, and cervical cancer), while subsequent administration of butyrate, a metabolite of vancomycin‐sensitive bacteria, reversed the antitumor effects.^115^ Nonetheless, few studies have evaluated the efficacy of antibiotics in brain tumors. Furthermore, antibiotic medications may do more harm than good due to imprecise targeting of bacteria and subsequent reduction in bacterial diversity. For example, studies in patients with lung and kidney cancers found that antibiotic administration coupled with immunotherapy shortened progression‐free and overall survival.^116^, ^117^

### 4.4 Bacteriophages

Bacteriophages, a class of prokaryotic viruses that have evolved specifically to infect and replicate within bacteria, can be designed to specifically target detrimental bacteria.^118^ One study showed that a bioinorganic hybrid bacteriophage could target and kill *Fusobacterium nucleatum*, which can increase immunosuppressive myeloid‐derived suppressor cells in the tumor microenvironment of colorectal cancer. As a result, the combination of *Fusobacterium nucleatum*‐binding M13 phage with silver nanoparticles led to a significant downregulation of myeloid‐derived suppressor cells and notably extended overall survival time in mouse models when coupled with checkpoint inhibitors or chemotherapies.^119^ Further studies are warranted to verify the therapeutic efficacy of bacteriophages for brain tumors.

### 4.5 Engineered microbiomes

The application of genetically engineered microbes, which have higher specificity than FMT has shown promising antitumor effects in preclinical models.^120^ Recent studies harnessed the targeting abilities of certain strains (*Salmonella* and *Clostridium*) as carriers for the delivery and induction of immune stimulants to delay tumor growth and metastasis in melanoma, renal cell carcinoma, and osteosarcoma.^121^ In addition, SYNB1891, designed based on the biology of *Escherichia coli*, has been modified to express the STING agonist cyclic adenosine diphosphate ribose (CADPR), thereby stimulating expression of IFNs and achieving antitumor effects in tumor‐bearing mouse models (melanoma, lymphoma, mammary carcinoma, and colon carcinoma).^122^ Further, another study showed that the engineered *Escherichia coli Nissle 1917* strain could colonize tumor sites, converting ammonia to L‐arginine, thereby increasing the intracellular concentration of L‐arginine in subcutaneous colonic cancer cells, which further triggered intra‐tumoral infiltration of CD4+ and CD8+ T cells, thus exerting synergistic antitumor effects when combined with anti‐PD‐L1.^123^ Collectively, engineered microbiomes show potential as an adjuvant therapy against a few kinds of tumors, and future research to evaluate their potency in brain tumors is merited.

### 4.6 Diet

Diet also constitutes a significant component of microbiota manipulation. Accumulating evidence indicates that changes to dietary regimens can rapidly alter gut microbial profiles.^124^ Meanwhile, dietary intervention also plays a role in the management of brain tumors. For example, ketogenic diets, comprising high‐fat, low‐carbohydrate, and adequate‐protein, can reduce seizure frequency by increasing GABA and glutamate levels in epilepsy patients.^125^ These findings are generalizable to diseases such as gliomas, with one pre‐clinical study detecting massive tumor cell death upon concurrent treatment of mice with calorically restricted ketogenic diet and a glutamine antagonist, without obvious host toxicity.^126^ Considering the close interaction between gut microbiota and diet patterns, the gut microbiota might serve as an intermediate between dietary intervention and brain tumors, although further research is required.

## 5 CONCLUSION

As technology develops, our knowledge of host‐microbiota interactions is rapidly expanding. Among these critical interactions, the gut‐brain axis has attracted interest from and offered novel insights to neuro‐oncologists. The mechanisms underlying the gut‐brain axis in brain tumors include immunological modulation, microbial metabolite‐mediated modulation, and direct invasion. Investigations into different types of brain tumors have shown deviations in the gastrointestinal microbial flora from the general population, indicating the involvement of the gut‐brain axis in the pathophysiological process. Further, emerging microbial interventions (FMT, probiotics, antibiotics and bacteriophage etc.) have begun to show therapeutic potential in the treatment of intracranial lesions, especially for inhibiting tumor progression. Thus, further efforts to decipher the alterations of gut‐brain axis in neuro‐oncology are warranted and will shed light on improving clinical management of brain tumors.

## AUTHOR CONTRIBUTIONS

BL and YCZ designed the manuscript. BL wrote the manuscript, incorporated the comments from all the co‐authors, and designed the figures of the manuscript. ZY, MW, and YCZ prepared the figures. ZC and RYG constructed the table. ZY, RYG, and YCZ edited the manuscript and provided valuable input for further improvement.

## CONFLICT OF INTEREST

The authors declare that they have no known competing financial interests or personal relationships that could have appeared to influence the work reported in this article.

## CONSENT FOR PUBLICATION

Not applicable.

## Data Availability

Data sharing is not applicable to this article as no new data were created or analyzed in this study.

## References

[1] Siegel RL , Miller KD , Jemal A . Cancer statistics, 2016. CA Cancer J Clin. 2016;66(1):7‐30. 26742998 10.3322/caac.21332

[2] Ostrom QT , Cioffi G , Waite K , Kruchko C , Barnholtz‐Sloan JS . CBTRUS statistical report: primary brain and other central nervous system tumors diagnosed in the United States in 2014‐2018. Neuro Oncol. 2021;23(12 Suppl 2):iii1‐iii105. 34608945 10.1093/neuonc/noab200 PMC8491279

[3] Touat M , Idbaih A , Sanson M , Ligon KL . Glioblastoma targeted therapy: updated approaches from recent biological insights. Ann Oncol. 2017;28(7):1457‐1472. 28863449 10.1093/annonc/mdx106 PMC5834086

[4] Human Microbiome Project C . Structure, function and diversity of the healthy human microbiome. Nature. 2012;486(7402):207‐214. 22699609 10.1038/nature11234 PMC3564958

[5] Vernocchi P , Del Chierico F , Putignani L . Gut microbiota profiling: metabolomics based approach to unravel compounds affecting human health. Front Microbiol. 2016;7:1144. 27507964 10.3389/fmicb.2016.01144 PMC4960240

[6] Cryan JF , Dinan TG . Mind‐altering microorganisms: the impact of the gut microbiota on brain and behaviour. Nat Rev Neurosci. 2012;13(10):701‐712. 22968153 10.1038/nrn3346

[7] Wang TY , Tao SY , Wu YX , et al. Quinoa reduces high‐fat diet‐induced obesity in mice via potential microbiota‐gut‐brain‐liver interaction mechanisms. Microbiol Spectr. 2022;10(3):e0032922. 35583337 10.1128/spectrum.00329-22 PMC9241864

[8] Fung TC , Olson CA , Hsiao EY . Interactions between the microbiota, immune and nervous systems in health and disease. Nat Neurosci. 2017;20(2):145‐155. 28092661 10.1038/nn.4476 PMC6960010

[9] Jing Y , Bai F , Wang L , et al. Fecal microbiota transplantation exerts neuroprotective effects in a mouse spinal cord injury model by modulating the microenvironment at the lesion site. Microbiol Spectr. 2022;10:e0017722. 35467388 10.1128/spectrum.00177-22 PMC9241636

[10] Vinithakumari AA , Padhi P , Hernandez B , et al. Clostridioides difficile infection dysregulates brain dopamine metabolism. Microbiol Spectr. 2022;10(2):e0007322. 35323033 10.1128/spectrum.00073-22 PMC9045323

[11] Wang H , Song W , Wu Q , et al. Fecal transplantation from db/db mice treated with sodium butyrate attenuates ischemic stroke injury. Microbiol Spectr. 2021;9(2):e0004221. 34612696 10.1128/Spectrum.00042-21 PMC8510264

[12] Belkaid Y , Hand TW . Role of the microbiota in immunity and inflammation. Cell. 2014;157(1):121‐141. 24679531 10.1016/j.cell.2014.03.011 PMC4056765

[13] Szefel J , Kruszewski WJ , Buczek T . Enteral feeding and its impact on the gut immune system and intestinal mucosal barrier. Prz Gastroenterol. 2015;10(2):71‐77. 26557936 10.5114/pg.2015.48997 PMC4631273

[14] Bene K , Varga Z , Petrov VO , Boyko N , Rajnavolgyi E . Gut microbiota species can provoke both inflammatory and tolerogenic immune responses in human dendritic cells mediated by retinoic acid receptor alpha ligation. Front Immunol. 2017;8:427. 28458670 10.3389/fimmu.2017.00427 PMC5394128

[15] Choi J , Kim BR , Akuzum B , Chang L , Lee JY , Kwon HK . T(REG)king from gut to brain: the control of regulatory T cells along the gut‐brain axis. Front Immunol. 2022;13:916066. 35844606 10.3389/fimmu.2022.916066 PMC9279871

[16] Pasciuto E , Burton OT , Roca CP , et al. Microglia require CD4 T cells to complete the fetal‐to‐adult transition. Cell. 2020;182(3):625‐640 e624. 32702313 10.1016/j.cell.2020.06.026 PMC7427333

[17] Zitvogel L , Galluzzi L , Viaud S , et al. Cancer and the gut microbiota: an unexpected link. Sci Transl Med. 2015;7(271):271ps1. 10.1126/scitranslmed.3010473 PMC4690201 25609166

[18] Bauer H , Horowitz RE , Levenson SM , Popper H . The response of the lymphatic tissue to the microbial flora. Studies on germfree mice. Am J Pathol. 1963;42:471‐483. 13966929 PMC1949649

[19] Mazmanian SK , Liu CH , Tzianabos AO , Kasper DL . An immunomodulatory molecule of symbiotic bacteria directs maturation of the host immune system. Cell. 2005;122(1):107‐118. 16009137 10.1016/j.cell.2005.05.007

[20] Atarashi K , Nishimura J , Shima T , et al. ATP drives lamina propria T(H)17 cell differentiation. Nature. 2008;455(7214):808‐812. 18716618 10.1038/nature07240

[21] Caballero S , Pamer EG . Microbiota‐mediated inflammation and antimicrobial defense in the intestine. Annu Rev Immunol. 2015;33:227‐256. 25581310 10.1146/annurev-immunol-032713-120238 PMC4540477

[22] Round JL , Mazmanian SK . Inducible Foxp3+ regulatory T‐cell development by a commensal bacterium of the intestinal microbiota. Proc Natl Acad Sci USA. 2010;107(27):12204‐12209. 20566854 10.1073/pnas.0909122107 PMC2901479

[23] Albulescu R , Codrici E , Popescu ID , et al. Cytokine patterns in brain tumour progression. Mediators Inflamm. 2013;2013:979748. 23864770 10.1155/2013/979748 PMC3707225

[24] Nellore A , Fishman JA . The microbiome, systemic immune function, and allotransplantation. Clin Microbiol Rev. 2016;29(1):191‐199. 26656674 10.1128/CMR.00063-15 PMC4771220

[25] Louveau A , Smirnov I , Keyes TJ , et al. Structural and functional features of central nervous system lymphatic vessels. Nature. 2015;523(7560):337‐341. 26030524 10.1038/nature14432 PMC4506234

[26] Quail DF , Joyce JA . The microenvironmental landscape of brain tumors. Cancer Cell. 2017;31(3):326‐341. 28292436 10.1016/j.ccell.2017.02.009 PMC5424263

[27] Yin J , Valin KL , Dixon ML , Leavenworth JW . The role of microglia and macrophages in CNS homeostasis, autoimmunity, and cancer. J Immunol Res. 2017;2017:5150678‐5150612. 29410971 10.1155/2017/5150678 PMC5749282

[28] Perry VH . Microglia. Microbiol Spectr. 2016;4:3. 10.1128/microbiolspec.MCHD-0003-2015 27337461

[29] Hong S , Dissing‐Olesen L , Stevens B . New insights on the role of microglia in synaptic pruning in health and disease. Curr Opin Neurobiol. 2016;36:128‐134. 26745839 10.1016/j.conb.2015.12.004 PMC5479435

[30] Thion MS , Garel S . On place and time: microglia in embryonic and perinatal brain development. Curr Opin Neurobiol. 2017;47:121‐130. 29080445 10.1016/j.conb.2017.10.004

[31] Erny D , Hrabe de Angelis AL , Jaitin D , et al. Host microbiota constantly control maturation and function of microglia in the CNS. Nat Neurosci. 2015;18(7):965‐977. 26030851 10.1038/nn.4030 PMC5528863

[32] Matcovitch‐Natan O , Winter DR , Giladi A , et al. Microglia development follows a stepwise program to regulate brain homeostasis. Science. 2016;353(6301):aad8670. 27338705 10.1126/science.aad8670

[33] Ding X , Zhou J , Zhao L , et al. Intestinal flora composition determines microglia activation and improves epileptic episode progress. Front Cell Infect Microbiol. 2022;12:835217. 35356535 10.3389/fcimb.2022.835217 PMC8959590

[34] Prosniak M , Harshyne LA , Andrews DW , et al. Glioma grade is associated with the accumulation and activity of cells bearing M2 monocyte markers. Clin Cancer Res. 2013;19(14):3776‐3786. 23741072 10.1158/1078-0432.CCR-12-1940

[35] Toney AM , Albusharif M , Works D , et al. Differential effects of whole red raspberry polyphenols and their gut metabolite urolithin a on neuroinflammation in BV‐2 microglia. Int J Environ Res Public Health. 2020;18(1):68. 33374120 10.3390/ijerph18010068 PMC7795536

[36] Haghikia A , Jorg S , Duscha A , et al. Dietary fatty acids directly impact central nervous system autoimmunity via the small intestine. Immunity. 2015;43(4):817‐829. 26488817 10.1016/j.immuni.2015.09.007

[37] Ooi YC , Tran P , Ung N , et al. The role of regulatory T‐cells in glioma immunology. Clin Neurol Neurosurg. 2014;119:125‐132. 24582432 10.1016/j.clineuro.2013.12.004

[38] Kingsbury C , Shear A , Heyck M , et al. Inflammation‐relevant microbiome signature of the stroke brain, gut, spleen, and thymus and the impact of exercise. J Cereb Blood Flow Metab. 2021;41(12):3200‐3212. 34427146 10.1177/0271678X211039598 PMC8669279

[39] McFarland BC , Hong SW , Rajbhandari R , et al. NF‐kappaB‐induced IL‐6 ensures STAT3 activation and tumor aggressiveness in glioblastoma. PLoS One. 2013;8(11):e78728. 24244348 10.1371/journal.pone.0078728 PMC3823708

[40] Zanotto‐Filho A , Goncalves RM , Klafke K , et al. Inflammatory landscape of human brain tumors reveals an NFkappaB dependent cytokine pathway associated with mesenchymal glioblastoma. Cancer Lett. 2017;390:176‐187. 28007636 10.1016/j.canlet.2016.12.015

[41] Sarkar A , Lehto SM , Harty S , Dinan TG , Cryan JF , Burnet PWJ . Psychobiotics and the manipulation of bacteria‐gut‐brain signals. Trends Neurosci. 2016;39(11):763‐781. 27793434 10.1016/j.tins.2016.09.002 PMC5102282

[42] Spiljar M , Merkler D , Trajkovski M . The immune system bridges the gut microbiota with systemic energy homeostasis: focus on TLRs, mucosal barrier, and SCFAs. Front Immunol. 2017;8:1353. 29163467 10.3389/fimmu.2017.01353 PMC5670327

[43] Wang Y , Nan X , Zhao Y , et al. Dietary supplementation of inulin ameliorates subclinical mastitis via regulation of rumen microbial community and metabolites in dairy cows. Microbiol Spectr. 2021;9(2):e0010521. 34494854 10.1128/Spectrum.00105-21 PMC8557905

[44] Xia J , Lv L , Liu B , et al. Akkermansia muciniphila ameliorates acetaminophen‐induced liver injury by regulating gut microbial composition and metabolism. Microbiol Spectr. 2022;10(1):e0159621. 35107323 10.1128/spectrum.01596-21 PMC8809353

[45] Braniste V , Al‐Asmakh M , Kowal C , et al. The gut microbiota influences blood‐brain barrier permeability in mice. Sci Transl Med. 2014;6(263):263ra158. 10.1126/scitranslmed.3009759 PMC4396848 25411471

[46] Nohr MK , Pedersen MH , Gille A , et al. GPR41/FFAR3 and GPR43/FFAR2 as cosensors for short‐chain fatty acids in enteroendocrine cells vs FFAR3 in enteric neurons and FFAR2 in enteric leukocytes. Endocrinology. 2013;154(10):3552‐3564. 23885020 10.1210/en.2013-1142

[47] Ratajczak W , Ryl A , Mizerski A , Walczakiewicz K , Sipak O , Laszczynska M . Immunomodulatory potential of gut microbiome‐derived short‐chain fatty acids (SCFAs). Acta Biochim pol. 2019;66(1):1‐12. 30831575 10.18388/abp.2018_2648

[48] Singh N , Thangaraju M , Prasad PD , et al. Blockade of dendritic cell development by bacterial fermentation products butyrate and propionate through a transporter (Slc5a8)‐dependent inhibition of histone deacetylases. J Biol Chem. 2010;285(36):27601‐27608. 20601425 10.1074/jbc.M110.102947 PMC2934627

[49] Nastasi C , Candela M , Bonefeld CM , et al. The effect of short‐chain fatty acids on human monocyte‐derived dendritic cells. Sci Rep. 2015;5:16148. 26541096 10.1038/srep16148 PMC4635422

[50] Wang G , Huang S , Wang Y , et al. Bridging intestinal immunity and gut microbiota by metabolites. Cell Mol Life Sci. 2019;76(20):3917‐3937. 31250035 10.1007/s00018-019-03190-6 PMC6785585

[51] Zelante T , Iannitti RG , Cunha C , et al. Tryptophan catabolites from microbiota engage aryl hydrocarbon receptor and balance mucosal reactivity via interleukin‐22. Immunity. 2013;39(2):372‐385. 23973224 10.1016/j.immuni.2013.08.003

[52] Cervantes‐Barragan L , Chai JN , Tianero MD , et al. Lactobacillus reuteri induces gut intraepithelial CD4(+)CD8alphaalpha(+) T cells. Science. 2017;357(6353):806‐810. 28775213 10.1126/science.aah5825 PMC5687812

[53] Sonner JK , Keil M , Falk‐Paulsen M , et al. Dietary tryptophan links encephalogenicity of autoreactive T cells with gut microbial ecology. Nat Commun. 2019;10(1):4877. 31653831 10.1038/s41467-019-12776-4 PMC6814758

[54] Westfall S , Caracci F , Zhao D , et al. Microbiota metabolites modulate the T helper 17 to regulatory T cell (Th17/Treg) imbalance promoting resilience to stress‐induced anxiety‐ and depressive‐like behaviors. Brain Behav Immun. 2021;91:350‐368. 33096252 10.1016/j.bbi.2020.10.013 PMC7986984

[55] Cuevas‐Sierra A , Ramos‐Lopez O , Riezu‐Boj JI , Milagro FI , Martinez JA . Diet, gut microbiota, and obesity: links with host genetics and epigenetics and potential applications. Adv Nutr. 2019;10(suppl_1):S17‐S30. 30721960 10.1093/advances/nmy078 PMC6363528

[56] Baj A , Moro E , Bistoletti M , Orlandi V , Crema F , Giaroni C . Glutamatergic signaling along the microbiota‐gut‐brain axis. Int J Mol Sci. 2019;20(6):1482. 30934533 10.3390/ijms20061482 PMC6471396

[57] Dang L , White DW , Gross S , et al. Cancer‐associated IDH1 mutations produce 2‐hydroxyglutarate. Nature. 2009;462(7274):739‐744. 19935646 10.1038/nature08617 PMC2818760

[58] Masui K , Tanaka K , Ikegami S , et al. Glucose‐dependent acetylation of Rictor promotes targeted cancer therapy resistance. Proc Natl Acad Sci USA. 2015;112(30):9406‐9411. 26170313 10.1073/pnas.1511759112 PMC4522814

[59] Wang Z , Chen G . Insights about circadian clock in glioma: from molecular pathways to therapeutic drugs. CNS Neurosci Ther. 2022;28(12):1930‐1941. 36066207 10.1111/cns.13966 PMC9627379

[60] Vallianatou T , Lin W , Bechet NB , et al. Differential regulation of oxidative stress, microbiota‐derived, and energy metabolites in the mouse brain during sleep. J Cereb Blood Flow Metab. 2021;41(12):3324‐3338. 34293940 10.1177/0271678X211033358 PMC8669215

[61] Lehnardt S , Lachance C , Patrizi S , et al. The toll‐like receptor TLR4 is necessary for lipopolysaccharide‐induced oligodendrocyte injury in the CNS. J Neurosci. 2002;22(7):2478‐2486. 11923412 10.1523/JNEUROSCI.22-07-02478.2002 PMC6758325

[62] Zhao J , Bi W , Xiao S , et al. Neuroinflammation induced by lipopolysaccharide causes cognitive impairment in mice. Sci Rep. 2019;9(1):5790. 30962497 10.1038/s41598-019-42286-8 PMC6453933

[63] Wang Y , Telesford KM , Ochoa‐Reparaz J , et al. An intestinal commensal symbiosis factor controls neuroinflammation via TLR2‐mediated CD39 signalling. Nat Commun. 2014;5:4432. 25043484 10.1038/ncomms5432 PMC4118494

[64] Engevik MA , Versalovic J . Biochemical features of beneficial microbes: foundations for therapeutic microbiology. Microbiol Spectr. 2017;5(5):5. 10.1128/microbiolspec.bad-0012-2016 PMC5873327 28984235

[65] El Aidy S , Kunze W , Bienenstock J , Kleerebezem M . The microbiota and the gut‐brain axis: insights from the temporal and spatial mucosal alterations during colonisation of the germfree mouse intestine. Benef Microbes. 2012;3(4):251‐259. 23234727 10.3920/BM2012.0042

[66] Neuman H , Debelius JW , Knight R , Koren O . Microbial endocrinology: the interplay between the microbiota and the endocrine system. FEMS Microbiol Rev. 2015;39(4):509‐521. 25701044 10.1093/femsre/fuu010

[67] Diaz Heijtz R , Wang S , Anuar F , et al. Normal gut microbiota modulates brain development and behavior. Proc Natl Acad Sci USA. 2011;108(7):3047‐3052. 21282636 10.1073/pnas.1010529108 PMC3041077

[68] Asano Y , Hiramoto T , Nishino R , et al. Critical role of gut microbiota in the production of biologically active, free catecholamines in the gut lumen of mice. Am J Physiol Gastrointest Liver Physiol. 2012;303(11):G1288‐G1295. 23064760 10.1152/ajpgi.00341.2012

[69] Divyashri G , Krishna G , Muralidhara PSG . Probiotic attributes, antioxidant, anti‐inflammatory and neuromodulatory effects of Enterococcus faecium CFR 3003: in vitro and in vivo evidence. J Med Microbiol. 2015;64(12):1527‐1540. 26450608 10.1099/jmm.0.000184

[70] Wang Y , Tong Q , Ma SR , et al. Oral berberine improves brain dopa/dopamine levels to ameliorate Parkinson's disease by regulating gut microbiota. Signal Transduct Target Ther. 2021;6(1):77. 33623004 10.1038/s41392-020-00456-5 PMC7902645

[71] Mitchell RW , On NH , Del Bigio MR , Miller DW , Hatch GM . Fatty acid transport protein expression in human brain and potential role in fatty acid transport across human brain microvessel endothelial cells. J Neurochem. 2011;117(4):735‐746. 21395585 10.1111/j.1471-4159.2011.07245.x

[72] Clarke G , Grenham S , Scully P , et al. The microbiome‐gut‐brain axis during early life regulates the hippocampal serotonergic system in a sex‐dependent manner. Mol Psychiatry. 2013;18(6):666‐673. 22688187 10.1038/mp.2012.77

[73] Liu WH , Chuang HL , Huang YT , et al. Alteration of behavior and monoamine levels attributable to lactobacillus plantarum PS128 in germ‐free mice. Behav Brain Res. 2016;298(Pt B):202‐209. 26522841 10.1016/j.bbr.2015.10.046

[74] Matsumoto M , Kibe R , Ooga T , et al. Cerebral low‐molecular metabolites influenced by intestinal microbiota: a pilot study. Front Syst Neurosci. 2013;7:9. 23630473 10.3389/fnsys.2013.00009 PMC3632785

[75] Janik R , Thomason LAM , Stanisz AM , Forsythe P , Bienenstock J , Stanisz GJ . Magnetic resonance spectroscopy reveals oral lactobacillus promotion of increases in brain GABA, N‐acetyl aspartate and glutamate. Neuroimage. 2016;125:988‐995. 26577887 10.1016/j.neuroimage.2015.11.018

[76] Bravo JA , Forsythe P , Chew MV , et al. Ingestion of Lactobacillus strain regulates emotional behavior and central GABA receptor expression in a mouse via the vagus nerve. Proc Natl Acad Sci USA. 2011;108(38):16050‐16055. 21876150 10.1073/pnas.1102999108 PMC3179073

[77] Mazzoli R , Pessione E . The neuro‐endocrinological role of microbial glutamate and GABA signaling. Front Microbiol. 2016;7:1934. 27965654 10.3389/fmicb.2016.01934 PMC5127831

[78] Olivares M , Schuppel V , Hassan AM , et al. The potential role of the dipeptidyl peptidase‐4‐like activity from the gut microbiota on the host health. Front Microbiol. 2018;9:1900. 30186247 10.3389/fmicb.2018.01900 PMC6113382

[79] Holzer P , Farzi A . Neuropeptides and the microbiota‐gut‐brain axis. Adv Exp Med Biol. 2014;817:195‐219. 24997035 10.1007/978-1-4939-0897-4_9 PMC4359909

[80] Kwon YH , Wang H , Denou E , et al. Modulation of gut microbiota composition by serotonin signaling influences intestinal immune response and susceptibility to colitis. Cell Mol Gastroenterol Hepatol. 2019;7(4):709‐728. 30716420 10.1016/j.jcmgh.2019.01.004 PMC6462823

[81] Le Guennec L , Coureuil M , Nassif X , Bourdoulous S . Strategies used by bacterial pathogens to cross the blood‐brain barrier. Cell Microbiol. 2020;22(1):e13132. 31658405 10.1111/cmi.13132

[82] Cummins J , Tangney M . Bacteria and tumours: causative agents or opportunistic inhabitants? Infect Agent Cancer. 2013;8(1):11. 23537317 10.1186/1750-9378-8-11 PMC3668256

[83] Nejman D , Livyatan I , Fuks G , et al. The human tumor microbiome is composed of tumor type‐specific intracellular bacteria. Science. 2020;368(6494):973‐980. 32467386 10.1126/science.aay9189 PMC7757858

[84] Zhao J , He D , Lai HM , et al. Comprehensive histological imaging of native microbiota in human glioma. J Biophotonics. 2022;15(4):e202100351. 34936211 10.1002/jbio.202100351

[85] Sampson JH , Gunn MD , Fecci PE , Ashley DM . Brain immunology and immunotherapy in brain tumours. Nat Rev Cancer. 2020;20(1):12‐25. 31806885 10.1038/s41568-019-0224-7 PMC7327710

[86] Kim H , Roh HS , Kim JE , Park SD , Park WH , Moon JY . Compound K attenuates stromal cell‐derived growth factor 1 (SDF‐1)‐induced migration of C6 glioma cells. Nutr Res Pract. 2016;10(3):259‐264. 27247721 10.4162/nrp.2016.10.3.259 PMC4880724

[87] Patrizz A , Dono A , Zorofchian S , et al. Glioma and temozolomide induced alterations in gut microbiome. Sci Rep. 2020;10(1):21002. 33273497 10.1038/s41598-020-77919-w PMC7713059

[88] Dono A , Patrizz A , McCormack RM , et al. Glioma induced alterations in fecal short‐chain fatty acids and neurotransmitters. CNS Oncologia. 2020;9(2):CNS57. 10.2217/cns-2020-0007 PMC7341178 32602743

[89] Jiang H , Zeng W , Zhang X , Pei Y , Zhang H , Li Y . The role of gut microbiota in patients with benign and malignant brain tumors: a pilot study. Bioengineered. 2022;13(3):7847‐7859. 35291914 10.1080/21655979.2022.2049959 PMC9208447

[90] Fan Y , Su Q , Chen J , Wang Y , He S . Gut microbiome alterations affect glioma development and Foxp3 expression in tumor microenvironment in mice. Front Oncol. 2022;12:836953. 35345443 10.3389/fonc.2022.836953 PMC8957261

[91] Li XC , Wu BS , Jiang Y , et al. Temozolomide‐induced changes in gut microbial composition in a mouse model of brain glioma. Drug des Devel Ther. 2021;15:1641‐1652. 10.2147/DDDT.S298261 PMC8071088 33907383

[92] Wang L , Li S , Fan H , et al. Bifidobacterium lactis combined with lactobacillus plantarum inhibit glioma growth in mice through modulating PI3K/AKT pathway and gut microbiota. Front Microbiol. 2022;13:986837. 36147842 10.3389/fmicb.2022.986837 PMC9486703

[93] D'Alessandro G , Antonangeli F , Marrocco F , et al. Gut microbiota alterations affect glioma growth and innate immune cells involved in tumor immunosurveillance in mice. Eur J Immunol. 2020;50(5):705‐711. 32034922 10.1002/eji.201948354 PMC7216943

[94] Dees KJ , Koo H , Humphreys JF , et al. Human gut microbial communities dictate efficacy of anti‐PD‐1 therapy in a humanized microbiome mouse model of glioma. Neurooncol Adv. 2021;3(1):vdab023. 33758825 10.1093/noajnl/vdab023 PMC7967908

[95] Walker AJ , Card T , Bates TE , Muir K . Tricyclic antidepressants and the incidence of certain cancers: a study using the GPRD. Br J Cancer. 2011;104(1):193‐197. 21081933 10.1038/sj.bjc.6605996 PMC3039809

[96] Aglae H , Philippe A , Croyal M , et al. Late‐stage glioma is associated with deleterious alteration of gut bacterial metabolites in mice. Metabolites. 2022;12(4):290. 35448477 10.3390/metabo12040290 PMC9028041

[97] Casanueva FF , Barkan AL , Buchfelder M , et al. Criteria for the definition of Pituitary Tumor Centers of Excellence (PTCOE): a Pituitary Society statement. Pituitary. 2017;20(5):489‐498. 28884415 10.1007/s11102-017-0838-2 PMC5606938

[98] Burton T , Le Nestour E , Neary M , Ludlam WH . Incidence and prevalence of acromegaly in a large US health plan database. Pituitary. 2016;19(3):262‐267. 26792654 10.1007/s11102-015-0701-2 PMC4858553

[99] Hacioglu A , Gundogdu A , Nalbantoglu U , et al. Gut microbiota in patients with newly diagnosed acromegaly: a pilot cross‐sectional study. Pituitary. 2021;24(4):600‐610. 33721175 10.1007/s11102-021-01137-4

[100] Nie D , Fang Q , Cheng J , et al. The intestinal flora of patients with GHPA affects the growth and the expression of PD‐L1 of tumor. Cancer Immunol Immunother. 2022;71(5):1233‐1245. 34647152 10.1007/s00262-021-03080-6 PMC9016060

[101] Lin B , Wang M , Gao R , et al. Characteristics of gut microbiota in patients with GH‐secreting pituitary adenoma. Microbiol Spectr. 2022;10(1):e0042521. 35019688 10.1128/spectrum.00425-21 PMC8754134

[102] Hu J , Yang J , Chen L , et al. Alterations of the gut microbiome in patients with pituitary adenoma. Pathol Oncol Res. 2022;28:1610402. 35991836 10.3389/pore.2022.1610402 PMC9385953

[103] Louis P , Flint HJ . Formation of propionate and butyrate by the human colonic microbiota. Environ Microbiol. 2017;19(1):29‐41. 27928878 10.1111/1462-2920.13589

[104] Allen AP , Hutch W , Borre YE , et al. Bifidobacterium longum 1714 as a translational psychobiotic: modulation of stress, electrophysiology and neurocognition in healthy volunteers. Transl Psychiatry. 2016;6(11):e939. 27801892 10.1038/tp.2016.191 PMC5314114

[105] Andersson H , Tullberg C , Ahrne S , et al. Oral administration of Lactobacillus plantarum 299v reduces cortisol levels in human saliva during examination induced stress: a randomized, double‐blind controlled trial. Int J Microbiol. 2016;2016:8469018‐8469017. 28101105 10.1155/2016/8469018 PMC5217173

[106] Messaoudi M , Lalonde R , Violle N , et al. Assessment of psychotropic‐like properties of a probiotic formulation (lactobacillus helveticus R0052 and Bifidobacterium longum R0175) in rats and human subjects. Br J Nutr. 2011;105(5):755‐764. 20974015 10.1017/S0007114510004319

[107] He Z , Cui BT , Zhang T , et al. Fecal microbiota transplantation cured epilepsy in a case with Crohn's disease: the first report. World J Gastroenterol. 2017;23(19):3565‐3568. 28596693 10.3748/wjg.v23.i19.3565 PMC5442093

[108] Liu L , Huh JR , Shah K . Microbiota and the gut‐brain‐axis: implications for new therapeutic design in the CNS. EBioMedicine. 2022;77:103908. 35255456 10.1016/j.ebiom.2022.103908 PMC8897630

[109] Sivan A , Corrales L , Hubert N , et al. Commensal Bifidobacterium promotes antitumor immunity and facilitates anti‐PD‐L1 efficacy. Science. 2015;350(6264):1084‐1089. 26541606 10.1126/science.aac4255 PMC4873287

[110] Mullard A . Oncologists tap the microbiome in bid to improve immunotherapy outcomes. Nat Rev Drug Discovery. 2018;17(3):153‐155. 29449710 10.1038/nrd.2018.19

[111] Matson V , Fessler J , Bao R , et al. The commensal microbiome is associated with anti‐PD‐1 efficacy in metastatic melanoma patients. Science. 2018;359(6371):104‐108. 29302014 10.1126/science.aao3290 PMC6707353

[112] Vetizou M , Pitt JM , Daillere R , et al. Anticancer immunotherapy by CTLA‐4 blockade relies on the gut microbiota. Science. 2015;350(6264):1079‐1084. 26541610 10.1126/science.aad1329 PMC4721659

[113] Liu A , Ma T , Xu N , et al. Adjunctive probiotics alleviates asthmatic symptoms via modulating the gut microbiome and serum metabolome. Microbiol Spectr. 2021;9(2):e0085921. 34612663 10.1128/Spectrum.00859-21 PMC8510161

[114] Keshavarz Azizi Raftar S , Ashrafian F , Yadegar A , et al. The protective effects of live and pasteurized Akkermansia muciniphila and its extracellular vesicles against HFD/CCl4‐induced liver injury. Microbiol Spectr. 2021;9(2):e0048421. 34549998 10.1128/Spectrum.00484-21 PMC8557882

[115] Uribe‐Herranz M , Rafail S , Beghi S , et al. Gut microbiota modulate dendritic cell antigen presentation and radiotherapy‐induced antitumor immune response. J Clin Invest. 2019;130(1):466‐479. 10.1172/JCI124332 PMC6934221 31815742

[116] Routy B , Le Chatelier E , Derosa L , et al. Gut microbiome influences efficacy of PD‐1‐based immunotherapy against epithelial tumors. Science. 2018;359(6371):91‐97. 29097494 10.1126/science.aan3706

[117] Derosa L , Hellmann MD , Spaziano M , et al. Negative association of antibiotics on clinical activity of immune checkpoint inhibitors in patients with advanced renal cell and non‐small‐cell lung cancer. Ann Oncol. 2018;29(6):1437‐1444. 29617710 10.1093/annonc/mdy103 PMC6354674

[118] Lim B , Zimmermann M , Barry NA , Goodman AL . Engineered regulatory systems modulate gene expression of human commensals in the gut. Cell. 2017;169(3):547‐558 e515. 28431252 10.1016/j.cell.2017.03.045 PMC5532740

[119] Dong X , Pan P , Zheng DW , Bao P , Zeng X , Zhang XZ . Bioinorganic hybrid bacteriophage for modulation of intestinal microbiota to remodel tumor‐immune microenvironment against colorectal cancer. Sci Adv. 2020;6(20):eaba1590. 32440552 10.1126/sciadv.aba1590 PMC7228756

[120] Zhou S , Gravekamp C , Bermudes D , Liu K . Tumour‐targeting bacteria engineered to fight cancer. Nat Rev Cancer. 2018;18(12):727‐743. 30405213 10.1038/s41568-018-0070-z PMC6902869

[121] Guo Y , Chen Y , Liu X , Min JJ , Tan W , Zheng JH . Targeted cancer immunotherapy with genetically engineered oncolytic salmonella typhimurium. Cancer Lett. 2020;469:102‐110. 31666180 10.1016/j.canlet.2019.10.033

[122] Leventhal DS , Sokolovska A , Li N , et al. Immunotherapy with engineered bacteria by targeting the STING pathway for anti‐tumor immunity. Nat Commun. 2020;11(1):2739. 32483165 10.1038/s41467-020-16602-0 PMC7264239

[123] Canale FP , Basso C , Antonini G , et al. Metabolic modulation of tumours with engineered bacteria for immunotherapy. Nature. 2021;598(7882):662‐666. 34616044 10.1038/s41586-021-04003-2

[124] Zhang S , Wu P , Tian Y , et al. Gut microbiota serves a predictable outcome of short‐term low‐carbohydrate diet (LCD) intervention for patients with obesity. Microbiol Spectr. 2021;9(2):e0022321. 34523948 10.1128/Spectrum.00223-21 PMC8557869

[125] Xie G , Zhou Q , Qiu CZ , et al. Ketogenic diet poses a significant effect on imbalanced gut microbiota in infants with refractory epilepsy. World J Gastroenterol. 2017;23(33):6164‐6171. 28970732 10.3748/wjg.v23.i33.6164 PMC5597508

[126] Mukherjee P , Augur ZM , Li M , et al. Therapeutic benefit of combining calorie‐restricted ketogenic diet and glutamine targeting in late‐stage experimental glioblastoma. Commun Biol. 2019;2:200. 31149644 10.1038/s42003-019-0455-x PMC6541653

